# *Notes from the Field:* Acquisition of Delamanid Under a Compassionate Use Program for Extensively Drug-Resistant Tuberculosis — United States, 2017

**DOI:** 10.15585/mmwr.mm6735a6

**Published:** 2018-09-07

**Authors:** Alfred A. Lardizabal, Anum N. Khan, Sapna Bamrah Morris, Neela D. Goswami

**Affiliations:** ^1^Global Tuberculosis Institute at Rutgers University, Brunswick, New Jersey; ^2^Rollins School of Public Health, Emory, Atlanta, Georgia; ^3^Division of Tuberculosis Elimination, CDC.

On April 10, 2017, a middle-aged man from Eastern Europe was evaluated at a hospital with cough, chest pain, weakness, and weight loss. A sputum sample was smear-positive for acid-fast bacilli (AFB), and chest radiograph and chest computerized tomography scan showed bilateral pulmonary, cavitary disease with vertebral involvement. He was given standard first-line therapy (HRZE): isoniazid, rifampin, pyrazinamide, and ethambutol. Among three of the patient’s family members evaluated as part of the contact investigation, his wife tested positive for tuberculosis (TB) infection via QuantiFERON-TB Gold In-Tube test and was treated for latent TB infection with 4 months of rifampin.*

Two weeks after initiating treatment, the patient developed a rash, and further TB medical consultation was sought. HRZE was discontinued, and he was started on levofloxacin and linezolid before being discharged from the hospital. Sputum was sent to CDC for Molecular Detection of Drug Resistance testing (https://www.cdc.gov/tb/topic/laboratory/default.htm), which identified mutations consistent with resistance to HRZE, fluoroquinolones, and the injectable aminoglycosides amikacin, kanamycin, and capreomycin. Drug susceptibility testing by 7H11 agar proportion at the National Jewish Health Mycobacteriology Laboratory (Denver, Colorado) confirmed resistance to HRZE, streptomycin, kanamycin, amikacin, capreomycin, ethionamide, and ciprofloxacin/ofloxacin, and reported susceptibility to cycloserine, para-aminosalicyclic acid (PAS), linezolid, clofazimine, and bedaquiline. Given the limited treatment options, the patient began a regimen of bedaquiline, clofazimine, linezolid, PAS, and cycloserine. In light of the extensively drug-resistant (XDR) disease and potential for limited drug tolerability, inclusion of delamanid, which is not yet approved by the Food and Drug Administration (FDA), was recommended by a CDC-funded TB Center of Excellence (TB COE) and the CDC’s Division of TB Elimination.

Delamanid, a nitro-imidazole, is a new anti-TB drug developed to address some of the adverse reactions and related adherence difficulties associated with currently available medications for treating drug-resistant TB. Multiple delamanid trials have evaluated safety, tolerability, and early bactericidal activity (*1*,*2*). Delamanid use, in combination with World Health Organization (WHO)–recommended drugs for drug-resistant TB, has shown improvement in treatment outcomes (*3*–*5*) and effectiveness against XDR TB, for which treatment options are limited (*6*). A U.S. physician, in conjunction with a TB COE and CDC’s Division of Tuberculosis Elimination, collaborated to acquire delamanid for this patient with XDR TB through a compassionate use program.

Per pharmaceutical manufacturer instructions, the proposed regimen and monitoring protocols were reviewed and approved by the European Respiratory Society/WHO Consilium, after which the treating physician was instructed to file an emergency investigational new drug application for single-patient expanded-access with FDA. Within 1 day of submission of the forms, FDA approved the emergency investigational new drug application for delamanid. The treating physician completed a company-sponsored 90-minute pharmacovigilance training webinar via teleconference. After requirements were fulfilled, medications were shipped. A 6-month supply of delamanid arrived within 5 days of application approval, and the patient was started on delamanid in late August. The patient’s initial symptoms, which included fatigue, anorexia, and cough, improved within the first month of treatment. His sputum smear converted to negative on August 22 and culture converted to negative October 31. The patient is continuing on a 24-month treatment plan at this time with continued clinical improvement and no reported adverse effects.

The process from application filing to delamanid initiation took 4 weeks. The pharmaceutical company provided clear instructions, contact information for concerns faced by the physician during treatment, and assistance completing FDA single-patient expanded-access forms. Physicians and state or local TB providers considering use of delamanid for their TB patients can seek guidance from CDC’s Division of Tuberculosis Elimination or their regional CDC-funded TB COE.^†^ Prompt access to this new anti-TB drug increased therapeutic options for this patient with XDR disease.

## Footnotes

* https://www.cdc.gov/tb/topic/treatment/ltbi.htm.

† CDC’s Division of Tuberculosis Elimination can be reached at 404-639-8120. Local CDC-funded TB COEs can be found at https://www.cdc.gov/tb/education/tb_coe/default.htm.
